# Joint-specific DNA methylation and transcriptome signatures in rheumatoid arthritis identify distinct pathogenic processes

**DOI:** 10.1038/ncomms11849

**Published:** 2016-06-10

**Authors:** Rizi Ai, Deepa Hammaker, David L. Boyle, Rachel Morgan, Alice M. Walsh, Shicai Fan, Gary S. Firestein, Wei Wang

**Affiliations:** 1 Department of Chemistry and Biochemistry, University of California San Diego, 9500 Gilman Dr., La Jolla, California 92093, USA; 2 Department of Medicine, School of Medicine, University of California San Diego, 9500 Gilman Dr., La Jolla, California 92093, USA; 3 Janssen Research and Development, 1400 McKean Road, Spring House, Pennsylvania 19477, USA; 4 School of Automation Engineering, University of Electronic Science and Technology of China, 2006 Xiyuan Avenue, Sichuan 611731, China

## Abstract

Stratifying patients on the basis of molecular signatures could facilitate development of therapeutics that target pathways specific to a particular disease or tissue location. Previous studies suggest that pathogenesis of rheumatoid arthritis (RA) is similar in all affected joints. Here we show that distinct DNA methylation and transcriptome signatures not only discriminate RA fibroblast-like synoviocytes (FLS) from osteoarthritis FLS, but also distinguish RA FLS isolated from knees and hips. Using genome-wide methods, we show differences between RA knee and hip FLS in the methylation of genes encoding biological pathways, such as IL-6 signalling via JAK-STAT pathway. Furthermore, differentially expressed genes are identified between knee and hip FLS using RNA-sequencing. Double-evidenced genes that are both differentially methylated and expressed include multiple *HOX* genes. Joint-specific DNA signatures suggest that RA disease mechanisms might vary from joint to joint, thus potentially explaining some of the diversity of drug responses in RA patients.

## Abstract (web summary)

Rheumatoid arthritis is an inflammatory disease that selectively affects different joints. Here the authors show that gene expression and DNA methylation patterns of fibroblast-like synoviocytes differ between hip and knee joints in patients with RA, thus providing epigenetic and transcriptomic evidence for this anatomic selectivity of inflammation.

## Graphical abstract

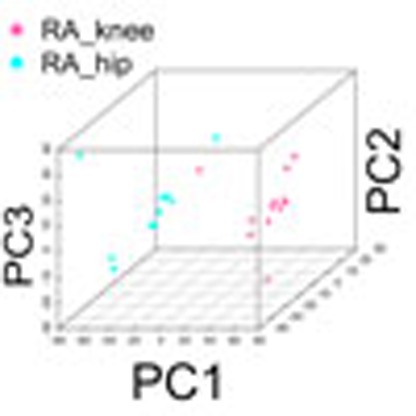

Rheumatoid arthritis (RA) therapy remains an unmet medical need, despite improvement^1^, in part due to the diversity of pathogenic pathways in RA^2^. In addition, the methods used to assess therapeutic response in clinical trials focus on total disease burden rather than synovitis in individual joints^3^,^4^,^5^,^6^. The distribution of RA is generally symmetrical and often evolves from the small joints of the hands and wrists to more diffuse involvement. However, there is no information as to why some joints are commonly inflamed (metacarpal phalangeal joints), some are uncommon (distal interphalangeal joints) and some are mainly affected in severe late disease (hips). We hypothesized that epigenetic patterns in the unique cells that line joints, namely fibroblast-like synoviocytes (FLS), contribute to differences in synovial inflammation and clinical response.

Growing evidence suggests that epigenetics has an important role in the pathogenesis of RA, which is an immune-mediated disease affecting diarthrodial joints^7^,^8^. We previously identified a DNA methylation signature that distinguishes RA FLS from osteoarthritis (OA) FLS^9^. These cells display a unique aggressive phenotype in RA and contribute to synovitis and matrix destruction through the production of small-molecule mediators, cytokines and matrix metalloproteinases^9^,^10^,^11^. By integrating our DNA methylation data with gene expression and genome-wide association study data, several potential drug targets were identified^12^. In addition, we showed differences of epigenomic signatures in early RA and long-standing RA suggesting plasticity in DNA methylation over time^13^.

Characteristic changes of synovitis, including synovial lining hyperplasia and sublining infiltration with mononuclear cells, are present in many joint types during RA. However, previous studies did not reveal significant differences in histology or cytokine expression between various RA joints^14^. However, those studies focused primarily on candidate gene approaches and used relatively insensitive techniques, such as immunohistochemistry. Alternative methods to assess spatially defined pathogenic mechanisms include quantification of epigenomic marks and transcriptomes using unbiased techniques. For example, DNA methylation has been profiled in various human tissues, and location-specific epigenomic patterns control tissue development and differentiation^15^,^16^,^17^,^18^,^19^,^20^,^21^.

As joint-specific pathogenic differences could influence response to therapy, here we evaluate DNA methylation and gene expression signatures in hips and knees. We first expand our RA and OA sample sets and confirm the RA epigenomic signature in FLS. We then compare DNA methylation and the transcriptome from FLS isolated from these two sites and identify joint-specific signatures and pathways. These data suggest that distinct mechanisms of disease in different joints could contribute to the pathogenesis of RA and variable responses to therapy.

## Results

### Confirmation and expansion of DNA methylation signature

We collected an independent set of 19 RA and 5 OA FLS from total joint replacement surgeries to complement our previous data set of 11 RA and 11 OA samples. Genome-wide analysis of DNA methylation by Infinium HumanMethylation450 BeadChip was used to examine methylation levels of 485,512 loci on the cultured FLS. We first confirmed the DNA methylation signature of RA defined in our previous work. In all, 2,956 differentially methylated loci (DMLs) were identified between 19 RA and 5 OA, and 72.5% overlapped with DMLs identified in the original data set^9^. After mapping DMLs to gene promoter region, 71.5% of 450 differentially methylated genes (DMGs) overlapped (Hypergeometric test; *P* value=4.26e-284; Supplementary Data 1). We then identified significantly enriched pathways related to the DMGs. In all, 13 out of 31 enriched pathways overlapped with pathways identified previously (Hypergeometric test; *P* value<0.05; Table 1 and Supplementary Table 1). Interestingly, multiple enriched pathways involved with inflammation, immunity and matrix destruction, strengthening the conclusion that the DMGs are related to mechanisms of disease.

**Figure 1:**
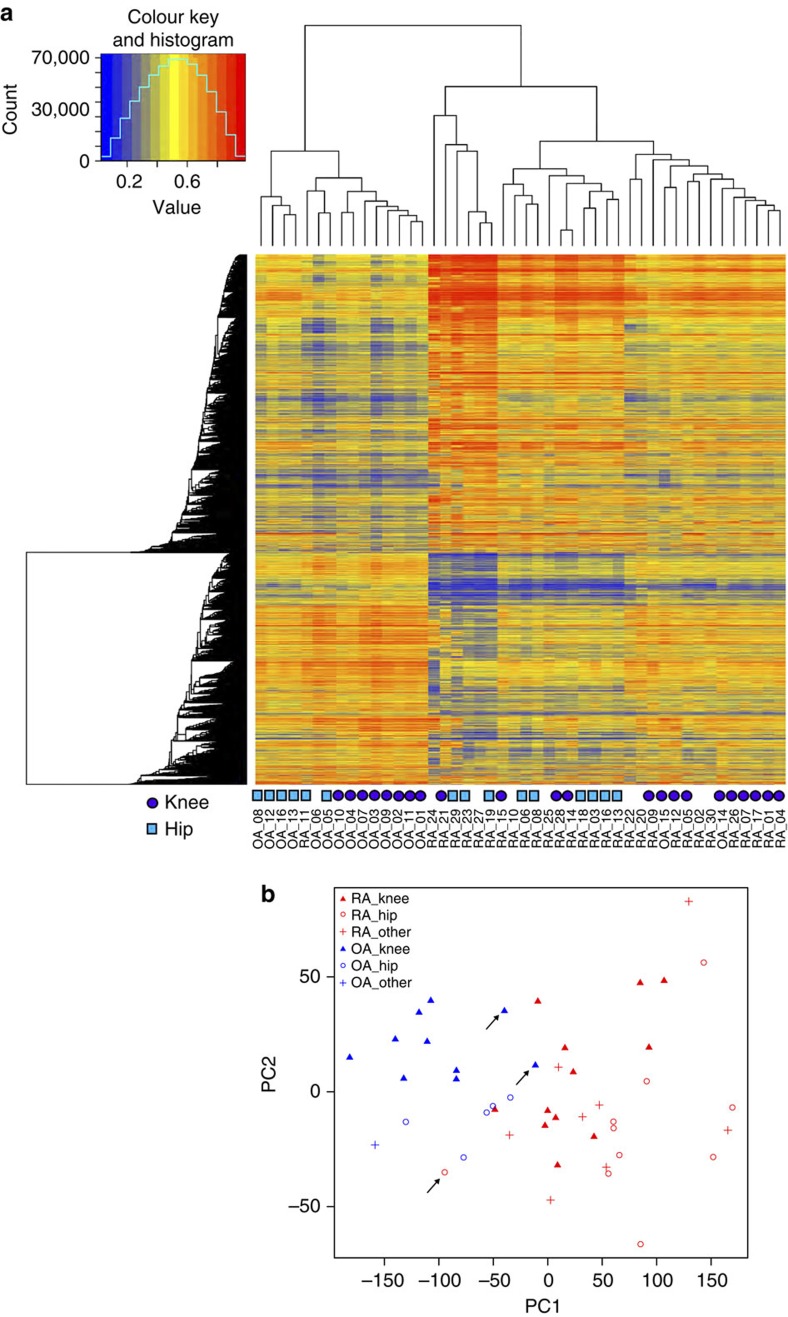
Unbiased clustering of a combinatory set of 30 RA and 16 OA. (**a**) Hierarchical clustering separated RA and OA into two groups (with three possible outliers: RA_11, OA_14 and OA_15). The FLS origins from knee and hip were labelled with purple circle and cyan square, respectively. The other unlabelled samples include FLS from other joints or where the source was unknown. (see Supplementary Table 3 for details). (**b**) PCA also divided RA and OA with the three possible outliers (arrows) at the edge of separation, indicating that these FLS lines were within the margins for their respective disease.

Next, we combined all of the samples (30 RA/16 OA) and identified 13,577 DMLs in 1,714 DMGs in the full data set. Sixty-seven significantly enriched pathways were identified, and 44 overlapped with the previous data, especially in pathways involving inflammation and immune responses (Supplementary Table 2). Using hierarchical clustering and principal component analysis (PCA), the 30 RA and 16 OA mainly segregated into two groups (Fig. 1a). The three possible outliers (RA_11 that clustered with OA samples and OA_14 and OA_15 that cluster with RA samples) shown in hierarchical clustering are positioned at edge of the RA and OA intersection in PCA plot and indicate that they are not outside the range for the specific disease (Fig. 1b).

### Joint-specific DNA methylation signatures in RA hip/knee FLS

**Figure 2:**
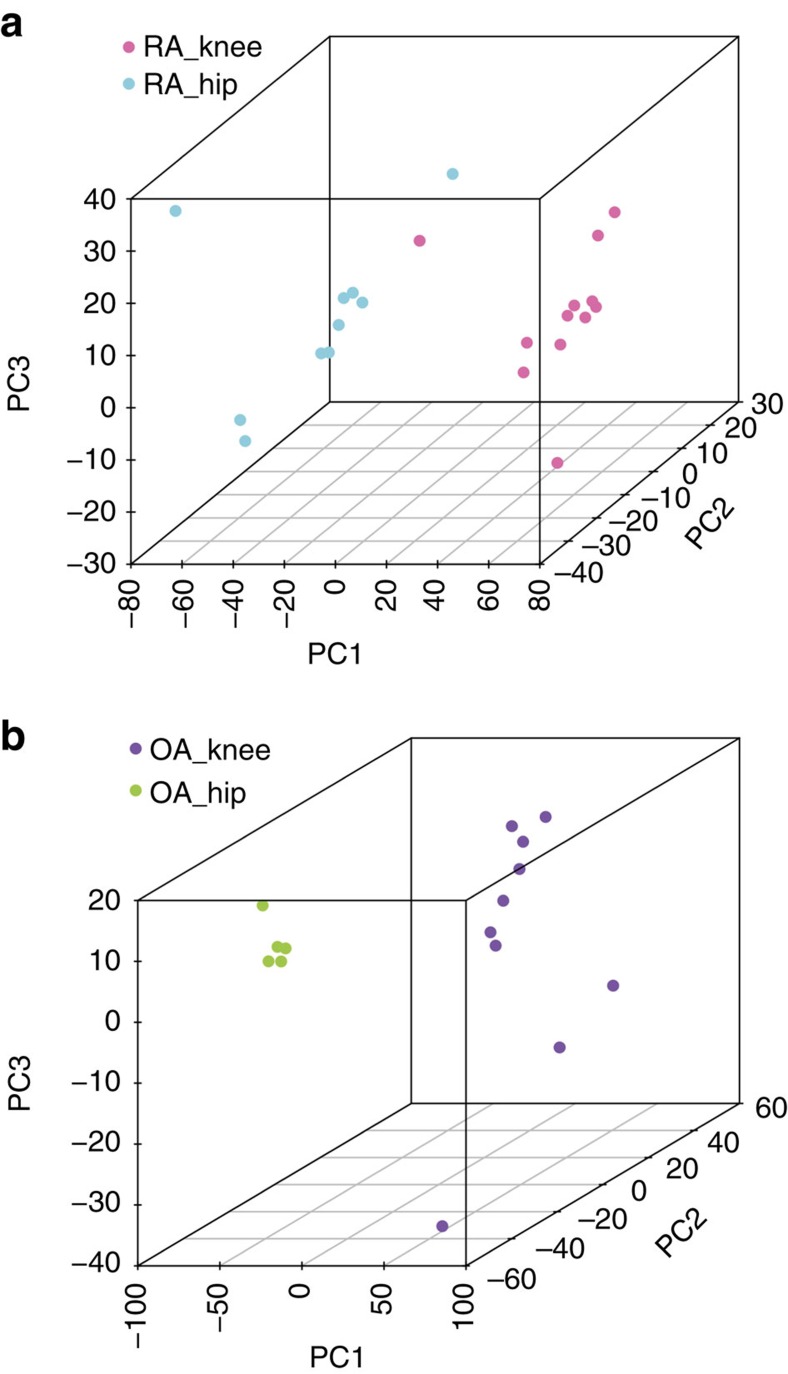
PCA of RA and OA joints by knee or hip methylation signatures. (**a**) Separation of RA knee and RA hip FLS using 3,739 DMLs. (**b**) Separation of OA knee and OA hip using the 6,416 DMLs. See text for description of how these DMLs were identified.

The increased number of samples in our confirmatory data set allowed us to perform separate clustering and PCA analyses focused different individual joint locations. Hips and knees are the most common joints requiring arthroplasty in RA and OA, and we therefore restricted further analysis to these FLS. Figure 1b shows aggregation of patient samples on the basis of joints of origin and suggest that the methylation pattern of these FLS could be distinguished (see also Supplementary Table 3 for details). To define the location-specific DNA methylation signatures in RA, we performed a Welch's *t*-test on the methylomes of 12 RA knee FLS and 10 RA hip FLS. A total of 3,739 DMLs (2,232 hypermethylated and 1,507 hypomethylated in RA knee FLS compared with hip) were identified (Supplementary Data 1). After mapping the 3,739 DMLs to gene promoter regions, 500 DMGs were identified with 285 hypermethylated, 210 hypomethylated and 5 with both hypermethylated and hypomethylated CpGs in RA knee FLS (Supplementary Data 1). Using 3,739 DMLs between RA knee and hip FLS, PCA separates RA knee from hip FLS with one RA knee FLS located at the edge of RA hip (Fig. 2a).

### Comparison of RA knee/hip differences to RA/OA differences

We then consider how the within-disease RA joint-specific differences compared with the overall differences between RA and OA. The number of RA knee/hip DMLs was 72.4% lower than the number RA/OA DMLs, suggesting that distinction between RA knee/hip is quantitatively less than the difference between RA and OA. Further analysis show that 391 (10.5%) of 3,739 RA knee/hip DMLs overlap with 13,577 RA/OA DMLs defined in our combined data set (30 RA and 16 OA).

### DNA methylation signatures in OA knee and OA hip joints

DNA methylation loci that differentiate OA knee and OA hip joints were then examined (Fig. 2b). A total of 6,416 DMLs (3,820 hypermethylated and 2,596 hypomethylated) in the promoter region of 814 DMGs (467 hypermethylated, 336 hypomethylated and 11 with both hypermethylated and hypomethylated DMLs) were identified between OA knee and hip FLS (Supplementary Data 1). 2,178 DMLs of the total 3,739 RA knee/hip DMLs overlap with the OA knee/hip DMLs. These overlapped DMLs could represent joint-specific methylation patterns that are independent of disease and instead reflect location-specific epigenetic marks, that is, a pattern that defines a hip FLS from a knee FLS.

### Disease-specific differences in knees and hips

Because the DNA methylation of 30 RA and 16 OA FLS were compared previously as a group, we also performed RA/OA comparisons within knees (12 RA to 10 OA) and within hips (10 RA to 5 OA), respectively (Supplementary Fig 1a,b). In all, 9,589 DMLs and 1,275 DMGs were identified between RA and OA knee FLS and 2,172 DMLs and 373 DMGs between RA and OA hip joint FLS. Of interest, 1,315 RA/OA DMLs overlapped between hips and knees. The majority of these DMLs (87.9% and 80.6% of between-disease DMLs in knees and hips, respectively) overlapped with the previously identified 13,577 RA/OA DMLs in combined data set (30 RA and 16 OA). The data indicate that the disease differences are still consistent in these two joints. The DMLs and DMGs are listed in the Supplementary Data 1.

### Joint-specific biological pathway identification

**Figure 3:**
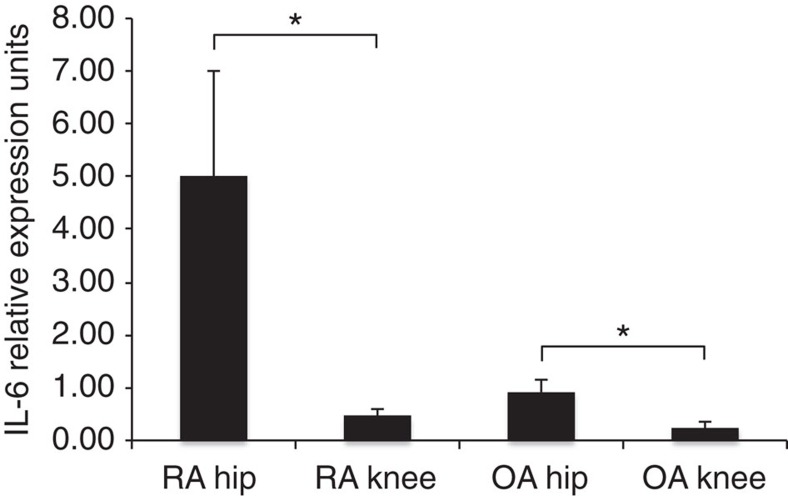
Synovial IL-6 expression levels using quantitative PCR. Intact synovial tissue was processed as described in the Methods and evaluated for IL-6 mRNA transcripts. Note that IL-6 gene expression is higher in RA than OA, and within-disease, higher in hip than knee. *P-values<0.05, calculated by two-tailed unpaired *t*-test with Welch's correction (*n*=13 RA hip, 22 RA knee, 13 OA hip, 15 OA knee). Data presented as mean±s.e.m. (see the Methods for details).

To examine disease-specific enriched pathways in different RA joints based on DNA methylation, we compared RA knee with hip FLS and then removed pathways potentially due to non-RA-associated influences because they were also found in OA FLS. Twelve RA knee and 10 OA knee FLS were combined and compared with 10 RA hip and 5 OA hip FLS combined. Optimally, normal FLS would be used for this analysis, but these samples are difficult to obtain and usually lack annotation to definitively exclude joint disease. As a surrogate, we used the RA–OA joint combination to help exclude some non-specific pathways. Eleven unique differentially methylated biological pathways (Hypergeometric test; *P* value<0.05) were found between RA knee and RA hip FLS (Table 2). Of particular interest are IL-6 signalling via JAK-STAT, which suggested that IL-6-related mechanisms might distinguish hips and knees. To test this hypothesis, IL-6 expression was measured in whole hip and knee synovia; IL-6 expression was 10.8-fold higher in RA hips than knees (two-tailed unpaired *t*-test with Welch's correction; *P*-value=0.042; *n*=13 RA hip, 22 RA knee, 13 OA hip, 15 OA knee; Fig. 3). Of interest, IL-6 expression was also higher in OA hip compared with OA knee FLS (two-tailed unpaired *t*-test with Welch's corrections; fold difference=3.72; *P*-value=0.027).

### Biological validation of joint-specific FLS function

**Figure 4:**
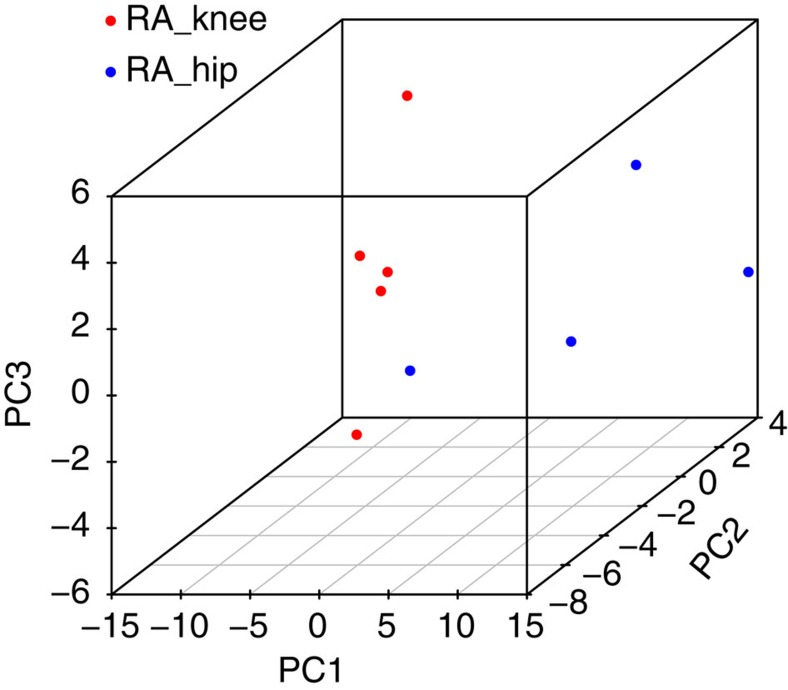
PCA of RA knee and RA hip FLS by differential gene expression analysis using RNA-seq. Distinct gene expression patterns were demonstrated that separated knee and hip transcriptomes. The PCA plot was generated using 107 DEGs between RA knee and hip FLS, with gene expression fold-change >2 and Benjamini–Hochberg adjusted *P*-values<0.05.

To determine whether the differences between hip and knee FLS extend to FLS function, we performed RNA sequencing (RNA-seq) experiments on 5 RA hip and 4 RA knee FLS lines. We identified 107 differentially expressed genes (DEGs) comparing RA hip and knee FLS (Supplementary Data 1). PCA shows that RA knee and RA hip FLS segregate from each other (Fig. 4). The enriched biological pathways related to the DEGs are shown in Table 3. Of interest, the ‘IL-6 signalling' pathway previously shown for DMGs was also observed for the RA knee and hip transcriptomes. Comparing 495 hyper- or hypo-DMGs, ten genes overlap (*SORBS2, HOXD3, LDLRAD4, EMB, HOXD10, HOXD9, HOXD-AS1, WNT5A, HOXB2, HAND2*). The overlap with genes associated with development, such as *HOX* and *WNT*, is especially striking. As a control, we also test pathways enrichment by randomly picking 10 groups of 107 human genes and performing pathway analysis. As expected, some of these gene sets reach statistical significance. However, in contrast to our RNA-seq data, the number of ‘enriched' pathways is much smaller for each gene set (usually five to ten) and the pathways are quite random and not related to immune function (data not shown).

### Potential therapeutic implications

**Figure 5:**
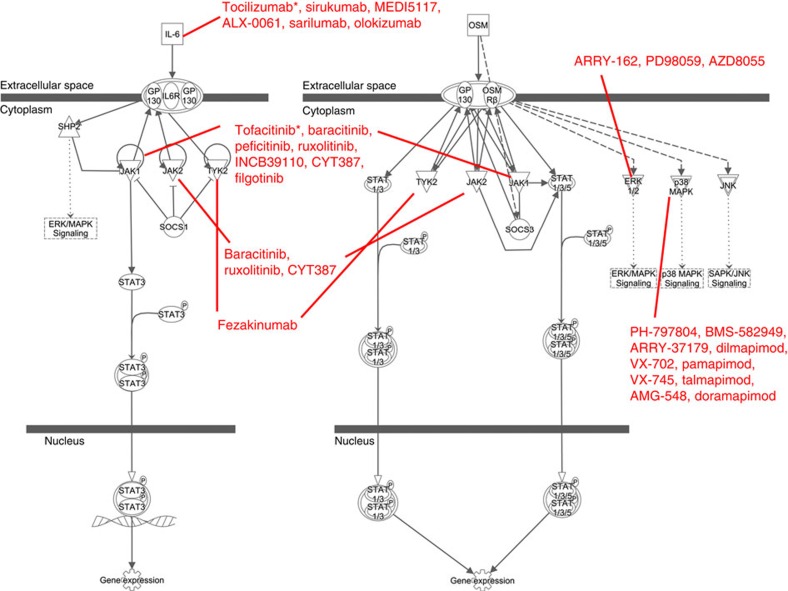
Representative joint-specific enriched biological pathway. The ingenuity pathway analysis ‘role of JAK family kinases in IL-6-type cytokine signalling' pathway is shown along with RA drug targets and anti-rheumatic drugs approved for use, in clinical trials or discontinued. Six RA drug targets were identified that have potential for differential responses between hips and knees, including IL-6, JAK1, JAK2, Tyk2, p38 MAPK and ERK1/2. Among the assigned RA drugs, tocilizumab and tofacitinib (labelled with *) that target IL-6 and JAKs, respectively, are currently approved for use in RA in the United States. The network was generated through the use of QIAGEN's Ingenuity Pathway Analysis (IPA®, QIAGEN Redwood City, www.qiagen.com/ingenuity)

To explore how differences in methylation and gene expression could affect joint-specific responses to drugs, we compiled a partial list of relevant anti-rheumatic drugs approved for use, in clinical trials or discontinued. This list of RA drugs was then assigned to the drug targets in joint-specific enriched RA knee or hip biological pathways (Table 2). Figure 5 shows one example of this analysis focused on the pathway ‘role of JAK family kinases in IL-6-type cytokine signalling'. Six RA drug targets were identified in the pathway, including IL-6, JAK1, JAK2, Tyk2, p38 MAPK and ERK1/2. Among the assigned RA drugs, tocilizumab and tofacitinib that target IL-6 and JAKs, respectively, are currently approved for use in RA. It is worth noting that several promising drugs with abundant pre-clinical data had only modest or no efficacy in RA, such as MEK and p38 inhibitors. Based on our data, we speculate that efficacy could have been under (or over) estimated if joint-specific pathways were not taken into account.

## Discussion

Our studies demonstrate joint location-specific epigenetic marks that could contribute to local pathogenic and developmental mechanisms. We focused on FLS for a variety of reasons, most notably because they display a unique aggressive phenotype in RA and contribute to cytokine production and joint damage. They also have not been targeted by new therapeutics and are a treatment opportunity that would not affect adaptive immunity^22^. Finally, FLS are a homogeneous population compared with the mixture of cells that populate the inflamed synovium and create complexity when trying to deconvolute data.

Using FLS and the expanded data set, we first confirmed the DNA methylation signature of RA defined in our previous study. We then showed that RA FLS have distinct DNA methylation and gene expression patterns based on the joint of origin. These data suggest that joints might differentially respond to highly targeted therapeutic agents and that this possibility should be considered in clinical trial design.

Our analysis has potential limitations, in part, because of the nature of arthroplasty surgery today. Hip and knee surgeries are the most common in RA and OA, which limits our ability to sample enough synovia from other joints. We also could not compare within-patient samples (that is, hip and knee from the same patient) because patients rarely have simultaneous arthroplasties. The use of cultured FLS introduces potential for the influence of culture conditions; current technology precluded the use of ‘primary' FLS. Nevertheless, the FLS methylation profile is stable despite prolonged culture and the aggressive FLS phenotype is maintained for months after isolating from synovial tissue^9^,^23^. Furthermore, hip and knee FLS were purified and grown the same way but still had very different methylomes and transcriptomes. Finally, our population is enriched for longstanding disease, which would be an advantage because it decreases the potential for epigenetic differences that might be based on duration of disease^13^. Despite these limitations, RA- and joint-specific genes and pathways were identified suggesting distinct pathogenic mechanisms can be identified even when potential disease-independent differences were taken into account.

The notion that all joints exhibit similar pathology in RA has been evaluated. For example, no significant differences in histology and cytokine expression in various joints were observed using immunohistochemistry^14^. The differences between our results and previous studies are likely due to the sensitivity of techniques and the use of unbiased rather than candidate gene approaches. One interesting corollary relates to the observation that FLS can potentially migrate from joint to joint^24^. Therefore, location-specific signatures suggest that local conditions could shape the epigenome after cells arrive.

We also compared the magnitude of RA hip/knee differences with RA/OA differences using computational methods. As expected, within-disease changes (for example, RA hip to RA knee) were less than the differences between RA and OA. Pathways and genes that are most likely related to hip and knee development because they were shared across diseases were estimated and subtracted from the RA hip and knee data to estimate RA-specific changes. The RA hip- or knee-specific pathways are related to inflammation and immune responses and support the notion that RA joints could have distinct pathogenic mechanisms.

Among the differences between hips and knees, we were struck by the preponderance of developmental genes that emerged in both transcriptomics and methylomics studies. The highly conserved *HOX* genes encode transcription factors of embryonic development that regulate limb morphogenesis and skeletal formation. During vertebrate embryo development, the *HOX* genes specify regional differences along the anterior–posterior axis^25^. The location-specific expression of *HOX* genes might contribute to defining joint-specific biology and function, and the impact on pathogenesis of disease is not fully understood. Differential expression of *HOX* genes in RA has been previously identified and supports the notion that the genes and pathways are not random^26^,^27^,^28^.

The clinical relevance of joint-specific pathways could be important when considering how drugs are tested in RA clinical trials. Current metrics do not distinguish which joints respond; instead, only the total number of swollen and tender joints is counted. Thus, investigators could potentially overlook site-specific responses or patterns. Hips are rarely evaluated in most clinical trial tools, such as the DAS28 (refs 29, 30), so a differential response in the hip would be missed entirely. Thus, clinical trialists had no method to determine whether individual joints differentially respond to targeted agents; to date, there was no particular reason to do so to consider this issue. Because our study found location-specific methylation and transcriptome signatures, it raises the possibility that asynchronous responses might occur with RA therapies. Although speculative, this could be an important consideration that in the context of clinical trials.

In conclusion, we have shown joint-specific differences in epigenetic imprinting and gene expression. Some of these differences could be due to the role of FLS in joint development. However, RA-specific differential gene expression and methylation pathways are also evident. The methylome and transcriptome differences between knees and hips in RA could potentially contribute to differential responses to highly targeted agents.

## Methods

### Synovial tissue and cell culture conditions

This study was approved by the Institutional Review Board of University of California, San Diego School of Medicine, and informed consent was obtained from all participants. Synovial tissue was obtained from patients with RA and OA at the time of total joint replacement or synovectomy, as previously described. In addition to the confirmatory samples, previously 11 RA and 11 OA (all female) reported synovial tissues were obtained with the same methods under the same Institutional Review Board approval.^9^,^10^ The diagnosis of RA conformed to American College of Rheumatology 2010 criteria^31^. The synovium was minced and incubated with 0.5 mg ml^−1^ collagenase type VIII (Sigma-Aldrich) in serum-free RPMI-1640 (Life Technologies) for 1 h at 37 °C, filtered, extensively washed and cultured in DMEM (Life Technologies) supplemented with 10% heat-inactivated fetal bovine serum (Gemini Bio Products) and supplements (penicillin, streptomycin, gentamicin and glutamine) in a humidified 5% CO_2_ atmosphere. Cells were allowed to adhere overnight, non-adherent cells were removed. Adherent FLSs were split at 1:3 when 70–80% confluent and used from passages 4 through 7 (ref. 32). Optimally, normal FLS would also be used for this analysis, but these samples are difficult to obtain and usually lack annotation to definitively exclude joint disease.

### FLS and patient phenotypes

Genomic DNA from RA (*n*=19; all female; average age 56) and OA (*n*=5; all female; average age 66) FLSs were isolated from patients requiring total joint replacements. Among the total 30 RA samples, 12 FLS were obtained from patient knees and 10 were from hips. Of 16 OA samples, 10 FLS were from knees and 5 were from hips. The origins of other joint samples are listed in Supplementary Table 3. Because some samples were de-identified, the joint of origin was not known. These samples were included in the overall assessment of RA compared with OA but not in the hip versus knee comparison.

### BeadChip analysis

DNA methylation level was measured using the Illumina Infinium HumanMethylation450 chip and processed with minfi package in R. The combined data set including 30 RAs and 16 OAs were normalized via the wateRmelon package in R (dasen function). CpGs with more than three detection *P*-values>0.01 were filtered out for OA and RA at each locus. The methylation level at individual locus was reported as *β* values, which varies from 0 (unmethylated) to 1 (fully methylated).

### DML and DMG identification

A Welch's *t*-test was performed to identify the significant DMLs. The *P*-values were subjected to a multiple testing correction and converted to *q* values using fdrtool package in R. To consider a DML, two conditions were used: (i) the differences of average *β* values were greater than 0.1 and (ii) *q* values were less than 0.05. DMLs located in the gene promoter regions were assigned to the corresponding genes, which were defined as DMGs. A promoter region of a gene is defined as −2,500 to 500 bps from the transcription start site. RefSeq genes, obtained from Genome Browser in University of California, Santa Cruz, were used to define the gene regions.

### Hierarchical clustering and PCA

Based on defined DML sets, hierarchical clustering was performed using *heatmap.2* function in R. Pearson correlation was used to calculate the distance matrix and *UPGMA* was adopted as the agglomeration method in hierarchical clustering. PCA was also performed to assess the relationships between different FLS types by *FactoMineR* (PCA function) in R.

### Unique RA knee and hip pathway enrichment analysis

To identify distinguished pathways, DMGs were mapped to Ingenuity pathway analysis (Qiagen; www.qiagen.com/ingenuity) and evaluated for enrichment. To identify the unique RA knee and hip pathways, (i) enriched biological pathways were combined using all DMGs, hypermethylated DMGs and hypomethylated DMGs between 12 RA knee and 10 RA hip; (ii) to avoid the RA-independent differences between knees and hips, knee FLS were represented by combining 12 RA-knee FLS and 10 OA-knee FLS, whereas hip FLS by combining 10 RA-hip FLS and 5 OA-hip FLS. All/hypermethylated/hypomethylated DMGs were identified and enriched biological pathways were combined; (iii) then, the unique pathways that distinguish RA knee and RA hip FLS were computed as by excluding pathways identified in (ii) from (i). To allow more enriched pathways included at each step, *P*-value cutoff of 0.1 was used. The final combined list only displayed unique pathways with *P*-values<0.05.

### Assigning RA drugs to uniquely enriched pathways

A list of current therapeutics to treat RA was obtained through the American College of Rheumatology website and the 2012 clinical practice guide^33^. Drugs in clinical trials were identified through the clinicaltrials.gov website and a literature search for RA clinical trials. Drugs were included if they are in use for, are currently under testing for or have been tried for RA. Ingenuity pathway analysis was used to analyse the pathways and cross-reference with the list of drug targets.

### RNA-seq data generation and processing

Total RNA was extracted and the quality of all samples was evaluated using an Agilent Bioanalyzer (Agilent). The samples had an average RNA integrity number of 9.4 with a minimum of 7.5. Sequencing libraries were prepared using TruSeq Stranded Total RNA RiboZero protocol from Illumina with an input of 400 ng RNA. Libraries were pooled and sequenced with an Illumina HiSeq 2000 with paired-end 100 bp flow cell. The average number of reads per sample was 171.8 million reads with a minimum of 154.5 million reads. Data were demultiplexed using Illlumina's CASAVA software. Raw read quality was evaluated using FastQC.

Adapter and low-quality bases below a quality score of 15 were trimmed from raw RNA-seq reads using Cutadapt. After trimming, reads with less than 30 bp were further discarded. The remaining reads were aligned to human reference genome hg19 using STAR (2.3.0) and assembled and quantified by HTSeq (0.5.4p5). DEGs were identified using DESeq2 package in R. To consider a DEG, twofold change of gene expression levels between RA knee and RA hip should be achieved and the Benjamini–Hochberg adjusted *P*-value is less than 0.05. For the following analysis, transcription levels were then converted to log2 of the normalized counts.

### Quantitative real-time PCR

Total RNA from synovial tissue was isolated using RNASTAT-60 and reverse transcribed (Applied Biosystems). The cDNA served as template for amplification by qPCR using TaqMan Gene Expression assays (StepOnePlus Instruments, Applied Biosystems). Data are presented as relative expression units, which uses the standard curve method and normalizes the Ct values to β-actin^34^. Primers were purchased from Life Technologies.

### Data availability

Data that support the findings of this study have been deposited in Gene Expression Omnibus with the accession codes GSE80071 and GSE80072. Data referenced in this study are available in Gene Expression Omnibus with the accession code GSE46364. All other relevant data are available from the corresponding authors.

## Additional information

**How to cite this article**: Ai, R. *et al*. Joint-specific DNA methylation and transcriptome signatures in rheumatoid arthritis identify distinct pathogenic processes. *Nat. Commun.* 7:11849 doi: 10.1038/ncomms11849 (2016).

## Supplementary Material

Supplementary Information Supplementary Figure 1 and Supplementary Tables 1-3.

Supplementary Data 1 DMLs identified between confirmatory set of 19 RA and 5 OA

## Figures and Tables

**Table 1:** Overlapped enriched methylation pathways between confirmatory and previous data sets.

**IPA pathways**	**Confirmtory set: 19 RA-5 OA**		**Previous set: 11 RA-11 OA**	
	**-log (*****P*****-value)**	**Ratio**	**-log (*****P*****-value)**	**Ratio**
Role of osteoblasts, osteoclasts and chondrocytes in rheumatoid arthritis	3.07	0.06	3.28	0.13
Atherosclerosis signalling	2.68	0.07	2.98	0.15
Hepatic fibrosis/hepatic stellate cell activation	2.35	0.05	4.31	0.15
Type II diabetes mellitus signalling	2.21	0.06	2.04	0.13
Role of macrophages, fibroblasts and endothelial cells in rheumatoid arthritis	2.04	0.04	2.22	0.11
Leukocyte extravasation signalling	1.50	0.04	2.28	0.12
ERK5 signalling	1.50	0.06	1.62	0.14
Angiopoietin signalling	1.45	0.06	1.54	0.14
DHA signalling	1.42	0.08	1.36	0.15
T helper cell differentiation	1.41	0.06	2.33	0.16
Human embryonic stem cell pluripotency	1.40	0.05	2.93	0.15
Relaxin signalling	1.39	0.05	1.50	0.11
Dendritic cell maturation	1.36	0.04	3.41	0.14

DHA, docosahexaenoic acid; IPA, Ingenuity pathway analysis; OA, osteoarthritis; Ratio, number of DMGs divided by the total number of genes in the pathway; RA, rheumatoid arthritis.

**Table 2:** Unique differentially methylated pathways between RA knee and hip FLS.

**Unique RA-knee versus RA-hip pathways**	**-log (*****P*****-value)**	**Ratio**	**Drug targets**	**Drugs**
IL-17A signalling in airway cells	1.98	0.08	IL-6, JAK1, JAK2, JAK3, p38 MAPK, IL-17A, NFkB, PI3K, MEK1/2	Tocilizumab*, sirukumab†, MEDI5117†, ALX-0061†, sarilumab†, olokizumab†, tofacitinib*, baracitinib†, peficitnib†, ruxolitinib†, INCB39110†, CYT387†, filgotinib†, PF-956980†, decernotinib†, R-348‡, PH-797804‡, BMS-582949‡, ARRY-371797‡, dilmapimod‡, VX-702‡, pamapimod‡, VX-745‡, talmapimod‡, AMG-548‡, doramapimod‡, secukinumab†, ixekizumab†, brodalumab†, iguratimod† (approved in China and Japan), CAL-263†, duvelisib‡, idelalisib‡, ARRY-162‡, PD98059‡, AZD8055‡
IL-22 signalling	1.87	0.13	JAK1, Tyk2, p38 MAPK, ERK1/2	Tofacitinib*, baracitinib†, peficitnib†, ruxolitinib†, INCB39110†, CYT387†, filgotinib†, fezakinumab‡, PH-797804‡, BMS-582949‡, ARRY-371797‡, dilmapimod‡, VX-702‡, pamapimod‡, VX-745‡, talmapimod‡, AMG-548‡, doramapimod‡, ARRY-162‡, PD98059‡, AZD8055‡
Role of JAK family kinases in IL-6-type cytokine signalling	1.82	0.12	IL-6, JAK1, JAK2, Tyk2, p38 MAPK, ERK1/2	Tocilizumab*, sirukumab†, MEDI5117†, ALX-0061†, sarilumab†, olokizumab†, tofacitinib*, baracitinib†, peficitnib†, ruxolitinib†, INCB39110†, CYT387†, filgotinib†, fezakinumab‡, PH-797804‡, BMS-582949‡, ARRY-371797‡, dilmapimod‡, VX-702‡, pamapimod‡, VX-745‡, talmapimod‡, AMG-548‡, doramapimod‡, ARRY-162‡, PD98059‡, AZD8055‡
p53 signalling	1.82	0.06	p38 MAPK, PI3K	PH-797804‡, BMS-582949‡, ARRY-371797‡, dilmapimod‡, VX-702‡, pamapimod‡, VX-745‡, talmapimod‡, AMG-548‡, doramapimod‡, CAL-263†, duvelisib‡, idelalisib‡
IL-17 signalling	1.77	0.07	IL-6, COX2, JAK1, JAK2, p38 MAPK, PI3K, iNOS, MEK1/2, CRP, CXCL10	Tocilizumab*, sirukumab†, MEDI5117†, ALX-0061†, sarilumab†, olokizumab†, NSAIDs*, tofacitinib*, baracitinib†, peficitnib†, ruxolitinib†, INCB39110†, CYT387†, filgotinib†, PH-797804‡, BMS-582949‡, ARRY-371797‡, dilmapimod‡, VX-702‡, pamapimod‡, VX-745‡, talmapimod‡, AMG-548‡, doramapimod‡, CAL-263, duvelisib‡, idelalisib‡, GW274150‡, ARRY-162‡, PD98059‡, AZD8055‡, ISIS-CRP‡, eldelumab‡
2-Amino-3-carboxymuconate semialdehyde degradation to glutaryl-CoA	1.68	1.00	NA	NA
Retinoate biosynthesis I	1.55	0.07	NA	NA
Inhibition of angiogenesis by TSP1	1.53	0.09	p38 MAPK	PH-797804‡, BMS-582949‡, ARRY-371797‡, dilmapimod‡, VX-702‡, pamapimod‡, VX-745‡, talmapimod‡, AMG-548‡, doramapimod‡
TGF-β signalling	1.46	0.06	p38 MAPK, MEK1/2	PH-797804‡, BMS-582949‡, ARRY-371797‡, dilmapimod‡, VX-702‡, pamapimod‡, VX-745‡, talmapimod‡, AMG-548‡, doramapimod‡, ARRY-162‡, PD98059‡, AZD8055‡
Acute phase response signalling	1.3	0.04	TNF, IL-1, GCR, IL-6, p38 MAPK, JAK2, CRP, C5a, mTOR, NFkB, PI3K, MEK1/2	Etanercept*, infliximab*, adalimumab*, certolizumab pegol*, golimumab*, SSR150106, anakinra*, canakinumab†, GSK1827771‡, corticosteroids*, tocilizumab*, sirukumab†, MEDI5117†, ALX-0061†, sarilumab†, olokizumab†, PH-797804‡, BMS-582949‡, ARRY-371797‡, dilmapimod‡, VX-702‡, pamapimod‡, VX-745‡, talmapimod‡, AMG-548‡, doramapimod‡, baracitinib†, ruxolitinib†, CYT387†, ISIS-CRP‡, NN 8209‡, NN 8210‡, eculizumab‡, MP-435‡, temsirolimus†, iguratimod† (approved in China and Japan), CAL-263†, duvelisib‡, idelalisib‡, ARRY-162‡, PD98059‡, AZD8055‡
Death receptor signalling	1.3	0.03	TNF, NFkB	Etanercept*, infliximab*, adalimumab*, certolizumab pegol*, golimumab*, SSR150106†, iguratimod† (approved in China and Japan)

FLS, fibroblast-like synoviocyte; NA, not applicable; RA, rheumatoid arthritis; NA, not applicable; Ratio, number of DMGs divided by the total number of genes in the pathway.

^*^Currently approved in the USA for RA.

^†^In clinical development at the time of publication.

^‡^Discontinued.

**Table 3:** Differentially expressed pathways between RA knee and hip FLS.

**IPA pathways**	**-log (*****P*****-value)**	**Ratio**
Coagulation system	3.44	0.10
Acute phase response signalling	2.19	0.03
Glutamate-dependent acid resistance	2.03	0.50
Hepatic fibrosis/hepatic stellate cell activation	2.01	0.02
IL-6 signalling	1.78	0.03
Protein citrullination	1.73	0.25
Glutamate degradation III (via 4-aminobutyrate)	1.64	0.20
Wnt/Ca+ pathway	1.58	0.04
Gαq signalling	1.53	0.02
Gap junction signalling	1.52	0.02
Tryptophan degradation to 2-amino-3- carboxymuconate semialdehyde	1.49	0.14
Salvage pathways of pyrimidine deoxyribonucleotides	1.43	0.13
Growth hormone signalling	1.41	0.03
Extrinsic prothrombin activation pathway	1.23	0.08
NAD biosynthesis II (from tryptophan)	1.23	0.08
Role of tissue factor in cancer	1.07	0.02
Type I diabetes mellitus signalling	1.06	0.02
Tryptophan degradation III (eukaryotic)	1.05	0.05
Sperm motility	1.02	0.02
Cellular effects of sildenafil	1.01	0.02

FLS, fibroblast-like synoviocyte; IPA, Ingenuity Pathway Analysis (see Methods); Ratio, number of DEGs divided by the total number of genes in the pathway; RA, rheumatoid arthritis.
