# Supplementary material for: Joint-specific DNA methylation and transcriptome signatures in rheumatoid arthritis identify distinct pathogenic processes
Source: Nat Commun. 2016 Jun 10;7:11849. doi: 10.1038/ncomms11849 (PMC4906396; doi:10.1038/ncomms11849)
Supplement: Supplementary Information — Supplementary Figure 1 and Supplementary Tables 1-3. [file ncomms11849-s1.pdf]

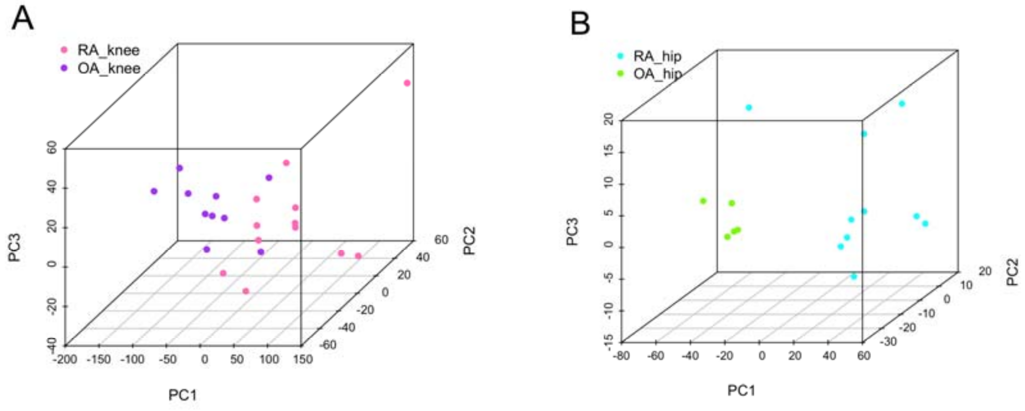

Supplementary Figure 1. PCA separated RA and OA FLS isolated from (a) knees and (b) hips by corresponding DMLs.

Supplementary Table 1. Enriched biological pathways identified between 19 RA and 5 OA in confirmatory set

Ingenuity Canonical Pathways	-log(p-value)	Ratio
Glycerol-3-phosphate Shuttle	3.44	1.00
Role of Osteoblasts, Osteoclasts and Chondrocytes in Rheumatoid Arthritis	3.07	0.06
Atherosclerosis Signaling	2.68	0.07
NAD Biosynthesis III	2.67	0.50
Circadian Rhythm Signaling	2.47	0.12
Glycerol Degradation I	2.46	0.40
NAD Salvage Pathway III	2.46	0.40
Hepatic Fibrosis / Hepatic Stellate Cell Activation	2.35	0.05
Acute Phase Response Signaling	2.30	0.05
NAD Biosynthesis from 2-amino-3-carboxymuconate Semialdehyde	2.29	0.33
Type II Diabetes Mellitus Signaling	2.21	0.06
NAD Salvage Pathway II	2.15	0.14
Role of Macrophages, Fibroblasts and Endothelial Cells in Rheumatoid Arthritis	2.04	0.04
NAD Phosphorylation and Dephosphorylation	1.83	0.20
Acyl-CoA Hydrolysis	1.75	0.18
TWEAK Signaling	1.61	0.09
NAD biosynthesis II (from tryptophan)	1.61	0.15
Stearate Biosynthesis I (Animals)	1.58	0.09
Leukocyte Extravasation Signaling	1.50	0.04
ERK5 Signaling	1.50	0.06
Angiopoietin Signaling	1.45	0.06
RAN Signaling	1.44	0.13
GABA Receptor Signaling	1.43	0.06
Alanine Degradation III	1.42	0.50
Alanine Biosynthesis II	1.42	0.50
Docosahexaenoic Acid (DHA) Signaling	1.42	0.08
T Helper Cell Differentiation	1.41	0.06
Human Embryonic Stem Cell Pluripotency	1.40	0.05
Relaxin Signaling	1.39	0.05
Dendritic Cell Maturation	1.36	0.04
Ephrin Receptor Signaling	1.33	0.04

Supplementary Table 2. Enriched biological pathways identified between 30 RA and 16 OA in combinatory set (44 pathways were overlapped with previous data)

Ingenuity Canonical Pathways	-log(p-value)	Ratio
**Overlapped Pathways**		
Axonal Guidance Signaling	2.96	0.12
Heme Degradation	2.83	0.75
Catecholamine Biosynthesis	2.83	0.75
ERK5 Signaling	2.74	0.19
Role of Osteoblasts, Osteoclasts and Chondrocytes in Rheumatoid Arthritis	2.70	0.13
Colorectal Cancer Metastasis Signaling	2.51	0.13
Acetate Conversion to Acetyl-CoA	2.46	0.60
LXR/RXR Activation	2.15	0.14
Angiopoietin Signaling	2.15	0.17
Type II Diabetes Mellitus Signaling	2.05	0.14
Hepatic Fibrosis / Hepatic Stellate Cell Activation	2.04	0.12
Agranulocyte Adhesion and Diapedesis	2.04	0.13
FXR/RXR Activation	2.01	0.14
NF-κB Signaling	1.92	0.12
Sphingosine-1-phosphate Signaling	1.92	0.14
Atherosclerosis Signaling	1.85	0.13
CXCR4 Signaling	1.84	0.13
Thyronamine and Iodothyronamine Metabolism	1.82	0.67
Thyroid Hormone Metabolism I (via Deiodination)	1.82	0.67
Relaxin Signaling	1.79	0.13
Leptin Signaling in Obesity	1.78	0.15
Sphingomyelin Metabolism	1.78	0.38
Granulocyte Adhesion and Diapedesis	1.75	0.12
Fcγ Receptor-mediated Phagocytosis in Macrophages and Monocytes	1.75	0.14
Cellular Effects of Sildenafil (Viagra)	1.72	0.13
eNOS Signaling	1.70	0.13
Tec Kinase Signaling	1.70	0.12
TR/RXR Activation	1.68	0.14
Docosahexaenoic Acid (DHA) Signaling	1.67	0.18
Protein Kinase A Signaling	1.66	0.10
Glucocorticoid Receptor Signaling	1.63	0.11
VDR/RXR Activation	1.62	0.14
Amyotrophic Lateral Sclerosis Signaling	1.61	0.13

P2Y Purigenic Receptor Signaling Pathway	1.60	0.13
Gustation Pathway	1.60	0.13
Human Embryonic Stem Cell Pluripotency	1.52	0.12
Glioblastoma Multiforme Signaling	1.44	0.12
Breast Cancer Regulation by Stathmin1	1.42	0.11
GPCR-Mediated Nutrient Sensing in Enteroendocrine Cells	1.41	0.13
Leukocyte Extravasation Signaling	1.35	0.11
Role of Macrophages, Fibroblasts and Endothelial Cells in Rheumatoid Arthritis	1.31	0.10
MSP-RON Signaling Pathway	1.31	0.15
Neuregulin Signaling	1.31	0.13
Gap Junction Signaling	1.30	0.11
**Unique pathways**		
Acute Phase Response Signaling	2.91	0.14
Complement System	2.41	0.22
Glycerol-3-phosphate Shuttle	2.27	1.00
Death Receptor Signaling	1.82	0.14
N-acetylglucosamine Degradation I	1.82	0.67
Ephrin A Signaling	1.65	0.17
ILK Signaling	1.62	0.12
RAN Signaling	1.59	0.25
Ephrin Receptor Signaling	1.57	0.12
Tumoricidal Function of Hepatic Natural Killer Cells	1.56	0.21
Sertoli Cell-Sertoli Cell Junction Signaling	1.55	0.12
NAD Biosynthesis III	1.54	0.50
N-acetylglucosamine Degradation II	1.54	0.50
Epithelial Adherens Junction Signaling	1.49	0.12
PEDF Signaling	1.48	0.14
Antiproliferative Role of TOB in T Cell Signaling	1.42	0.19
Thyroid Hormone Metabolism II (via Conjugation and/or Degradation)	1.42	0.19
Antioxidant Action of Vitamin C	1.38	0.13
NAD Salvage Pathway III	1.34	0.40
Citrulline-Nitric Oxide Cycle	1.34	0.40
Glycerol Degradation I	1.34	0.40
Corticotropin Releasing Hormone Signaling	1.31	0.12
Intrinsic Prothrombin Activation Pathway	1.30	0.18

Supplementary Table 3. Joint origins and patient information of 30 RA and 16 OA FLS

**FLS**	**Sex**	**Age**	**Joint location**	**FLS**	**Sex**	**Age**	**Joint location**
RA_01	F	57	knee	RA_24	F	NA	NA
RA_02	F	67	wrist	RA_25	F	42	ankle
RA_03	F	65	hip	RA_26	F	58	knee
RA_04	F	55	knee	RA_27	F	25	NA
RA_05	F	52	knee	RA_28	F	71	knee
RA_06	F	44	hip	RA_29	F	61	hip
RA_07	F	53	knee	RA_30	NA	NA	NA
RA_08	F	39	hip	OA_01	F	58	knee
RA_09	F	64	knee	OA_02	F	83	knee
RA_10	F	48	NA	OA_03	F	69	knee
RA_11	F	45	hip	OA_04	F	54	knee
RA_12	F	79	knee	OA_05	F	55	hip
RA_13	F	74	hip	OA_06	F	NA	NA
RA_14	F	73	knee	OA_07	F	53	knee
RA_15	F	68	knee	OA_08	F	83	hip
RA_16	F	44	hip	OA_09	F	57	knee
RA_17	F	64	knee	OA_10	F	80	knee
RA_18	F	46	hip	OA_11	F	76	knee
RA_19	F	19	hip	OA_12	F	64	hip
RA_20	F	67	elbow	OA_13	F	63	hip
RA_21	F	68	knee	OA_14	F	74	knee
RA_22	F	70	elbow	OA_15	F	56	knee
RA_23	F	24	hip	OA_16	F	72	hip
